# Oxaliplatin: pre-clinical perspectives on the mechanisms of action, response and resistance

**DOI:** 10.3332/ecancer.2009.153

**Published:** 2009-09-24

**Authors:** RN Seetharam, A Sood, S Goel

**Affiliations:** Department of Oncology, Montefiore Medical Center/Albert Einstein Cancer Center, 111 E 210th St, Bronx, NY 10467, USA

## Abstract

Oxaliplatin is a third-generation platinum compound that has shown a wide range of anti-tumour activity in metastatic cancer and in multiple cell lines. It contains a diaminocyclohexane carrier ligand and is one of the least toxic platinum agents. In the past decade, the use of oxaliplatin for the treatment of colorectal cancer has become increasingly popular because neither cisplatin nor carboplatin demonstrate significant activity. Similar to cisplatin, oxaliplatin binds to DNA, leading to GG intra-strand crosslinks. Oxaliplatin differs from its parent compounds in its mechanisms of action, cellular response and development of resistance, which are not fully understood. Like most chemotherapeutic agents, efficacy of oxaliplatin is limited by the development of cellular resistance. ERCC1 (excision repair cross-complementation group 1) mediated nucleotide excision repair pathway appears to be the major pathway involved in processing oxaliplatin, because the loss of mismatch repair does not lead to oxaliplatin resistance. Recent findings support the involvement of many genes and different pathways in developing oxaliplatin resistance. This mini-review focuses on the effects of oxaliplatin treatment on cell lines with special emphasis on colorectal cell lines.

## Introduction

Colorectal cancer is the third leading cause of cancer-related mortality in men and women in the United States. It is estimated that 146,970 men and women will be diagnosed with, and 49,920 will die, of this cancer in 2009 [1]. The last three decades have witnessed a significant amount of basic research on platinum coordination complexes, leading to the pre-clinical screening of several thousand new molecules, of which only a few have entered clinical development. Although platinum drugs have a broad range of activity against malignant tumours, they are particularly active against germ cell tumours and epithelial ovarian cancer. In addition, they play a primary role in the treatment of small cell and non-small-cell lung, cervical, head and neck, colorectal and bladder cancer [2]. The platinum drugs such as cisplatin, carboplatin and oxaliplatin are used to treat a broad range of cancers; however, in most cases, their efficacy is limited by the development of resistance [3]. Due to this, the primary objective of researchers working in this area has been to identify compounds with superior efficacy, reduced toxicity, lack of cross-resistance or improved pharmacological characteristics as compared with the parent compound, cisplatin. Oxaliplatin (trans-L-1,2-diamino cyclohexane oxalatoplatinum) is a third generation platinum compound and the first platinum-based compound to show efficacy in the treatment of colorectal cancer [4] and approved for therapy as a front-line agent [5]. The intracellular targets and mechanisms of action/resistance of oxaliplatin differ from that of its predecessors, cisplatin and carboplatin. It is important to note that oxaliplatin is more active in colon cells [6], and that cisplatin-resistant cell lines are sensitive to oxaliplatin [7, 8].

## Intracellular targets and mechanisms of action

Oxaliplatin and cisplatin are structurally distinct, but form the same types of adducts at the same sites on DNA [9–13]. In physiological conditions, oxaliplatin forms DNA adducts that are not at dynamic equilibrium [14]. Upon entering the cell, oxaliplatin first forms a transient monoadduct and then forms a stable diadduct, by mostly binding to the N(7) site of the guanine residues [15]. Intra-strand adducts are most abundant, and if not repaired, will block both DNA replication and transcription. Although platinum adducts can form inter-strand crosslinks by DNA–protein interaction, the proteinase resistant crosslinks are usually less than 1% of the total platinum adducts [16].

Oxaliplatin belongs to 1,2-diaminocyclohexane (DACH) carrier ligand family, whereas cisplatin and carboplatin belong to cis-diammine. There are some differences between compounds belonging to these families.

1. Bulkiness: DACH-Pt-DNA ligands are bulkier and more hydrophobic than cis-diammine-Pt-DNA and, perhaps, therefore, they are more effective in inhibiting DNA synthesis and are superior cytotoxic compounds [17].
2. Bond constraint: N–Pt–N bond angle is more constrained for DACH-Pt-DNA adducts than for cis-diammine-Pt-DNA adducts [18]. This might lead to slower mono-adduct to di-adduct conversion of DACH-Pt-DNA, leading to less stable adducts.
3. Computer modelling: the modelling indicated that this ring protrudes directly outward into and fills much of the narrowed major groove of the bound DNA, forming a markedly altered and less polar major groove in the area of the adduct. The differences in the structure of the adducts produced by cisplatin and oxaliplatin are consistent with the observation that they are differentially recognized by the DNA mismatch repair system, cisplatin being more easily recognized [11]. A detailed kinetic analysis of the insertion and extension steps of dNTP incorporation in the vicinity of the adduct shows that both DNA polymerase beta (pol beta) and DNA polymerase eta (pol eta) catalyse trans-lesion synthesis past oxaliplatin-GG adducts with greater efficiency than past cisplatin-GG adducts [19].

## Oxaliplatin processing

Mismatch repair proteins, DNA damage-recognition proteins and trans-lesion DNA polymerases discriminate between Pt-GG adducts containing cis-diammine ligands (formed by cisplatin and carboplatin) and trans-RR-diaminocyclohexane ligands (formed by oxaliplatin) [19,20]. It is known that mismatch repair proteins, such as MutS and hMSH2 bind to cisplatin, but not to oxaliplatin adducts [21]. Loss of mismatch repair produces low levels of resistance to cisplatin but not oxaliplatin [22]. So, nucleotide excision repair pathway appears to be the major pathway involved in the processing of oxaliplatin.

Low levels of XPA, a protein involved in making a nick at the third end of the platinum adduct, in the tests tumour cell lines is sufficient to explain their poor ability to remove platinum adducts from DNA [23,24].

## Nucleotide excision repair

ERCC1 and ERCC2 (xeroderma pigmentosa—XPD) are the two major genes involved in this pathway. It has been previously shown that the expression levels of the ERCC1 gene can significantly affect the ability of the drug to influence survival in patients with colon cancer [25].

**Figure 1:**
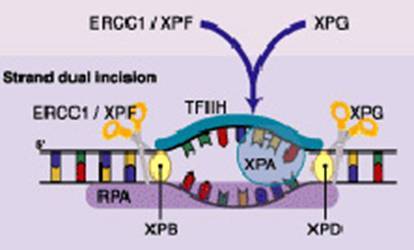
DNA strand dual incision, 5′ incision by ERCC1-XPF heterodimer is 22 nucleotide from lesion and 3′ incision by XPG is six nucleotides from the lesion

The nucleotide excision repair (NER) reaction is carried out by a multi-enzyme complex and involves a stepwise process of recognition, incision, excision, repair synthesis and ligation [26,27]. ERCC1 along with XPF forms a critical heterodimer of the NER pathway because of its damage recognition creation of nick 5′ to the lesion [26, 28–35]. XPF-ERCC1 is also known to be involved in recombinational DNA repair and in the repair of inter-strand crosslinks [36]. Figure 1 shows simplified steps in NER pathway.

While there are indications that the relative ERCC1 mRNA level is a good marker for NER activity in human cancer cells, it is unclear whether expression of this gene has any relationship to other pathways of DNA repair [37]. In a study of 50 patients with ERCC1 gene expression ≤ 4.9 × 10(−3) (40 of 50 patients) had a median survival time of 10.2 months, compared with 1.9 months for patients with ERCC1 expression greater than 4.9 × 10(−3) (p < .001) [25].

## Cellular response

There have been several studies on the cellular response of oxaliplatin in different types of cancer cell lines, sometimes suggesting contrasting results. In a study involving four cancer cell lines, ovarian and an inherently cisplatin-resistant colon (HT-29), ERCC1 mRNA levels measured after exposure to oxaliplatin for 20 hours were higher than in the control—the A2780 (ovarian) cell line [8]. Further, it was shown that, relative to cisplatin, a lower intracellular concentration and fewer DNA-Pt adducts are sufficient for oxaliplatin to exert its cytotoxicity [8]. Oxaliplatin is also capable of altering the voltage-gated sodium channels, thereby inducing both acute and chronic toxicity [38]. Another group studied the combination of irinotecan and oxaliplatin in HCT-8, a colorectal cancer cell line, and xenograft models and observed that ERCC1 expression was unregulated on exposure to oxaliplatin. Addition of irinotecan abrogated this effect, with the potential for synergy between the two drugs by the inhibition of DNA repair and increased cytotoxicity of the platinum [39]. In another study, it was shown that siRNA knockdown of ERCC1 expression resulted in sensitivity to oxaliplatin in the HeLa S3 (cervical cancer) cells [40].

Cytotoxicity of oxaliplatin on a panel of six colon cell lines *in vitro* showed that glutathione and glutathione S-transferase activity were not correlated to oxaliplatin cytotoxicity. Further, the expression of ERCC1 and XPA (xeroderma pigmentosum group A) demonstrated that ERCC1 expression was predictive of oxaliplatin sensitivity [41]. When DNA microarray analysis was used to analyse the transcriptional profile of resistant HCT116 colorectal cancer cells that were treated with oxaliplatin or 5-fluorouracil (5-FU), bioinformatic analyses identified sets of genes that were constitutively dysregulated in drug-resistant cells and transiently altered following acute exposure of parental cells to drug. This leads to the proposition that these genes may represent molecular signatures of sensitivity to oxaliplatin and 5-FU [42].

## Resistance

The existing body of literature suggests that the rate of NER may have a major impact on the emergence of resistance and normal tissue tolerance to platinum drugs [43]. DNA adducts are differentially recognized by a number of cellular proteins. For example, mismatch repair proteins and some damage-recognition proteins bind to cisplatin-GG adducts with higher affinity than to oxaliplatin-GG adducts, and this differential recognition of cisplatin- and oxaliplatin-GG adducts is thought to contribute to the differences in cytotoxicity and tumour range of cisplatin and oxaliplatin [19]. Elevation of glutathione mediated by gamma-glutamyl transpeptidase has also been shown to be a mechanism of oxaliplatin resistance [8]. Oxaliplatin-resistance may also involve multiple other pathways like down-regulation of pyruvate kinase M2 [44], altered mitochondrial-mediated apoptosis [45] and phosphoinositide-3- kinase (PI3K)/Akt activation [46]. DNA microarray studies suggest the involvement of large number of genes in developing oxaliplatin resistance [42,47].

## Conclusion and outlook

Although platinum drugs are one of the most widely used anti-cancer agents, the outcome of the treatment depends upon the drug resistance. Oxaliplatin has been shown to exhibit broad spectrum anti-tumour activity including a subset of cisplatin resistant cell lines. Pre-clinical studies have shown that ERCC1 gene expression plays critical role in the effectiveness of oxaliplatin treatment. Ongoing research will lead to the better understanding of the association between the expression levels of DNA excision repair genes and the response to oxaliplatin treatment. The goal of the ongoing research is to lead to the development of more effective compounds in this class.
